# Use of the kidney failure risk equation: a regional retrospective primary care cohort study in England

**DOI:** 10.3399/BJGP.2025.0490

**Published:** 2026-03-24

**Authors:** Stuart Stewart, Philip A Kalra, Evangelos Kontopantelis, Thomas Blakeman, George Tilston, Smeeta Sinha

**Affiliations:** 1 Centre for Primary Care and Health Services Research, University of Manchester, Manchester, UK; 2 Donal O’Donoghue Renal Research Centre, Northern Care Alliance NHS Foundation Trust, Salford Royal Hospital, Salford, UK; 3 University of Manchester, Manchester, UK; 4 National Institute for Health and Care Research Greater Manchester Patient Safety Research Collaboration, University of Manchester, Manchester, UK

**Keywords:** chronic renal insufficiency, population health, primary health care, risk prediction, renal replacement therapy, general practice

## Abstract

**Background:**

Chronic kidney disease (CKD) is associated with increased risk of death and progression to kidney failure requiring renal replacement therapy (RRT). Predicting those at greatest risk of RRT is essential for effective clinical care. The kidney failure risk equation (KFRE) is useful for predicting risk of RRT but does not consider the competing risk of death.

**Aim:**

To measure the association between KFRE scores and probability of death versus RRT; and to quantify the number of patients with a KFRE score >5%.

**Design and setting:**

Retrospective cohort study conducted from 2018 to 2023 using the primary care Greater Manchester Care Record.

**Method:**

A mixed descriptive and regression analyses. Multinomial regression was used to measure the association between KFRE score categories (<5%, 5%–20%, and >20%) and relative risk/probability of death and RRT.

**Results:**

In general, for the KFRE category of <5%: probability of RRT was 0.1% (95% confidence interval [CI] = 0.1 to 0.2) and death was 11.9% (95% CI = 11 to 13); for the KFRE category 5%–20%: probability of RRT was 2% (95% CI = 1 to 3) and death was 23% (95% CI = 20 to 27); and for the KFRE category of >20%: probability of RRT was 14% (95% CI = 8 to 23) and death was 29% (95% CI = 25 to 36). On average, 11% of patients with CKD stages 3–5 had a KFRE score >5% and could be eligible for referral to nephrology.

**Conclusion:**

The probability of death was generally greater than RRT across KFRE categories — useful for clinicians to consider in shared decision making and management. An estimate of all patients potentially eligible for referral to nephrology is useful for care delivery and provision of nephrology services.

## How this fits in

Determining kidney failure risk in chronic kidney disease is recommended in National Institute of Health and Care Excellence guidelines using the four-variable 5-year kidney failure risk equation (KFRE). However, the KFRE does not consider the competing risk of death. The authors show that the risk of death is often much greater than kidney failure. However, Black and Asian patients with a KFRE score >20% were at greater risk of kidney failure requiring renal replacement therapy than death. Current guidelines do not discuss ethnicity or competing risk of death as factors to guide referrals; however, doing so may improve quality and equity of care.

## Introduction

Chronic kidney disease (CKD) is a prevalent condition in primary care affecting ~10% of adults worldwide^1,2^ and predicted to be the fifth leading cause of life years lost by 2040.^1^ A major challenge for GPs is managing the complex interplay and interconnected risk of complications such as acute kidney injury, cardiovascular events, kidney failure requiring renal replacement therapy (RRT), and death.^3–5^

Addressing one aspect of this problem, GPs can use the kidney failure risk equation (KFRE) introduced in National Institute for Health and Care Excellence (NICE) CKD guidelines^6^ in 2021 to predict a patient’s 5-year risk of RRT using the patient’s age, sex, estimated glomerular filtration rate (eGFR), and urine albumin-to-creatinine ratio (uACR).

However, the KFRE does not consider the competing risk of death that may exceed RRT for different subgroups of patients.^7^ Furthermore, under NICE guidance,^6^ patients with a KFRE score of 5% may be considered for referral to nephrology^6^ yet there are no population-level estimates of how many patients are captured by this threshold.

Addressing these gaps is crucial because it would help GPs better navigate discussions with patients about their true risks, help prioritise conservative care versus concerted risk-factor management, and potentially reduce unnecessary referrals, while an accurate estimate of patients who could be referred could help shape the provision of clinical services.

This research therefore aimed to evaluate the association between KFRE scores (<5%, 5%–20%, and >20%) and probability of death versus RRT; and also to quantify the number of patients with a KFRE score >5% who could be referred to nephrology.

## Method

### Study design and participants

This was a retrospective observational cohort study using the Greater Manchester Care Record (GMCR; study reference: GMCR-RQ041) from 1 March 2018 to 1 August 2023. The GMCR pools de-identified electronic health record (EHR) data of 2.85 million citizens across 433 Greater Manchester general practices (99.7% of practices).^8^

Study participants included adult patients with CKD stages 3–5 defined using eGFR and uACR according to NICE guidelines.^6^ Patients who had no creatinine/eGFR and uACR results during the study period, or had diagnostic codes indicating dialysis or kidney transplant, were excluded. Patients with dementia or palliative care diagnostic codes were also excluded to minimise the inclusion of patients with a substantially increased risk of death. Four analytic cohorts were created:

- a cross-sectional CKD stages 3–5 cohort used to describe the overall CKD population;
- a CKD stages 3–5 cohort with uACR measured and therefore a calculable KFRE score;
- a CKD stages 3–5 cohort without uACR measured; and
- a multinomial regression subcohort — used to measure the association of KFRE score categories on probabilities of RRT versus death.

### Procedures

Outcomes included death (all-cause mortality) and RRT during the study period, indicated by diagnostic codes (for kidney transplant and dialysis), across a 5–6-year follow-up period. The index year was 2018. All eGFRs were recalculated using the CKD-EPI 2021 equation,^9^ and the latest eGFR was chosen for patients with multiple eGFRs within a year. All creatinine results with an acute kidney injury (AKI) code were excluded. Predictor variables included the four-variable UK KFRE^10^ modelled as categories (<5%, 5%–20%, and >20%), sex, age group, ethnicity, body mass index (BMI), socioeconomic status, measured using indices of multiple deprivation (IMD) deciles — a measure of geographical area level deprivation at a low geographical level of approximately 1600 people, and comorbidity diagnostic codes at study start (see Supplementary Tables S1 and S2).

### Statistical analyses

Descriptive statistics were used to summarise the study cohort; continuous variables are reported as means and standard deviations (SDs), and categorical variables presented as counts and percentages. Multinomial regression was used to quantify the relative risk (expressed as a relative risk ratio [RRR]) of outcomes across KFRE categories (<5%, 5%–20%, and >20%) and other independent variables over a 5–6-year follow-up period. Patients with missing BMI group, IMD, and ethnicity data were excluded. A *P*-value <0.05 was considered statistically significant. Data cleaning and statistical analyses were performed using R/RStudio (version 4.1.0).

## Results

Across all years, there were 109 543 patients with CKD stages 3–5; of these, 65 801 (60.1%) had at least one uACR measured during the study period and therefore a calculable KFRE score at least once (Table 1). Conversely, 43 742 patients (39.9%) with CKD stages 3–5 did not have uACR measured during the study period. There were 30 197 (27.6%) patients with CKD stages 3–5 and a measured uACR in 2018 or 2019 used in the multinomial regression analyses.

**Table 1.** Cohort clinical and demographic summary: patients with chronic kidney disease stages 1–3

Characteristic	Cumulative annual cross-sectional cohort, *n* (%),^a^ *N =* 109 543	Cumulative annual cohort with at least one calculable KFRE score, *n* (%),^a^ *N* = 65 801	Cumulative annual cohort without uACR during study, *n* (%),^a^ *N* = 43 742	Multinomial regression cohort (2018 and 2019), ***n* (%**),^a^ ***N*** **= 30 197**
**Sex, male**	60 987 (55.7)	38 199 (58.1)	22 788 (52.1)	19 666 (65.1)
**Age, mean, years (SD)**	72.4 (11.7)	72.3 (11.1)	72.5 (12.7)	74.1 (10.4)
**BMI**				
<18.5	1360 (1.2)	594 (0.9)	766 (1.8)	273 (0.9)
18.5–24.9	24 336 (22.2)	13 156 (20.0)	11 180 (25.6)	6178 (20.5)
25.0–29.9	40 412 (36.9)	24 446 (37.2)	15 966 (36.5)	11 651 (38.6)
30.0–39.9	34 416 (31.4)	22 692 (34.5)	11 724 (26.8)	10 501 (34.8)
≥40.0	4950 (4.5)	3361 (5.1)	1589 (3.6)	1408 (4.7)
Missing	4069 (3.7)	1552 (2.4)	2517 (5.8)	186 (0.6)
**Ethnicity**				
White or White British	86 406 (78.9)	51 962 (79.0)	34 444 (78.7)	23 481 (77.8)
Asian or Asian British	7178 (6.6)	5014 (7.6)	2164 (4.9)	2361 (7.8)
Black or Black British	2985 (2.7)	1874 (2.8)	1111 (2.5)	900 (3.0)
Mixed	769 (0.7)	443 (0.7)	326 (0.7)	183 (0.6)
Other ethnic groups	10 239 (9.3)	5513 (8.4)	4726 (10.8)	2816 (9.3)
Missing	1966 (1.8)	995 (1.5)	971 (2.2)	456 (1.5)
**IMD deciles**				
1 (most deprived)	22 823 (20.8)	14 168 (21.5)	8655 (19.8)	6534 (21.6)
2	15 237 (13.9)	9455 (14.4)	5782 (13.2)	4393 (14.5)
3	12 443 (11.4)	7545 (11.5)	4898 (11.2)	3433 (11.4)
4	8737 (8.0)	5239 (8.0)	3498 (8.0)	2360 (7.8)
5	8825 (8.1)	5243 (8.0)	3582 (8.2)	2394 (7.9)
6	6770 (6.2)	3887 (5.9)	2883 (6.6)	1681 (5.6)
7	8955 (8.2)	5264 (8.0)	3691 (8.4)	2468 (8.2)
8	10 417 (9.5)	6174 (9.4)	4243 (9.7)	2809 (9.3)
9	8610 (7.9)	5211 (7.9)	3399 (7.8)	2487 (8.2)
10 (least deprived)	6708 (6.1)	3603 (5.5)	3105 (7.1)	1631 (5.4)
Missing	18 (0.02)	12 (0.02)	6 (0.01)	7 (0.02)
**Diagnoses**				
Diabetes	32 981 (30.1)	28 761 (43.7)	4220 (9.6)	15 783 (52.3)
Hypertension	69 576 (63.5)	45 144 (68.6)	24 432 (55.9)	22 220 (73.6)
Systemic lupus erythematosus	305 (0.3)	179 (0.3)	126 (0.3)	83 (0.3)
Gout	12 228 (11.2)	7881 (12.0)	4347 (9.9)	4351 (14.4)
Non-alcoholic fatty liver disease	1642 (1.5)	1180 (1.8)	462 (1.1)	526 (1.7)
Myeloma	223 (0.2)	120 (0.2)	103 (0.2)	73 (0.2)
Osteoporosis	8167 (7.5)	4393 (6.7)	3774 (8.6)	1940 (6.4)
Coronary heart disease	21 663 (19.8)	14 198 (21.6)	7465 (17.1)	7584 (25.1)
Heart failure	7783 (7.1)	4842 (7.4)	2941 (6.7)	2705 (9.0)
Peripheral arterial disease	4729 (4.3)	3170 (4.8)	1559 (3.6)	1808 (6.0)
Stroke	7466 (6.8)	4516 (6.9)	2950 (6.7)	2405 (8.0)
Transient ischaemic attack	5415 (4.9)	3273 (5.0)	2142 (4.9)	1705 (5.6)
Autosomal dominant polycystic kidney disease	1244 (1.1)	752 (1.1)	492 (1.1)	409 (1.4)
Glomerulonephritis	564 (0.5)	299 (0.5)	265 (0.6)	146 (0.5)
Kidney stones	3927 (3.6)	2077 (3.2)	1850 (4.2)	886 (2.9)
Vasculitis	962 (0.9)	482 (0.7)	480 (1.1)	180 (0.6)
Acute kidney injury	16 128 (14.7)	10 121 (15.4)	6007 (13.7)	4564 (15.1)
Depression	32 625 (29.8)	19 356 (29.4)	13 269 (30.3)	8353 (27.7)
Schizophrenia	2166 (2.0)	1113 (1.7)	1053 (2.4)	482 (1.6)
Bipolar disorder	883 (0.8)	466 (0.7)	417 (1.0)	203 (0.7)
Eating disorder	1553 (1.4)	913 (1.4)	640 (1.5)	445 (1.5)
Self-harm and suicidal ideation	3445 (3.1)	1954 (3.0)	1491 (3.4)	761 (2.5)

^a^Unless otherwise specified. Missing data excluded from final multinomial regression analyses. Diagnoses based on those recorded in EHR at study start. BMI = body mass index. EHR = electronic health record. IMD = Index of Multiple Deprivation. KFRE = kidney failure risk equation. SD = standard deviation. uACR = urine albumin-to-creatinine ratio.

Male sex was more prevalent across all cohorts: 55.7% (*n* = 60 987) in the annual cross-sectional cohort; 58.1% (*n* = 38 199) in the KFRE subcohort; 52.1% (*n* = 22 788) in the no uACR cohort; and 65.1% (*n* = 19 666) in the regression subcohort. Mean age was 72.4 years (SD 11.7) in the annual cross-sectional cohort and similar across each cohort. Most patients within each cohort were in either the overweight or obese category. Considering the annual cross-sectional cohort, the majority of patients were of White ethnicity (78.9%; *n* = 86 406), 6.6% (*n* = 7178) of Asian ethnicity, 2.7% (*n* = 2985) of Black ethnicity, and 9.3% (*n* = 10 239) of other ethnic groups. More than 50% of all patients across each cohort lived in the four most deprived IMD deciles, with the highest proportion within each cohort (~20%) living in the most deprived decile. The five most prevalent comorbidities diagnosed at study start were hypertension (63.5%; *n* = 69 576), diabetes (30.1%; *n* = 32 981), depression (29.8%; *n* = 32 625), coronary heart disease (CHD) (19.8%; *n* = 21 663), and AKI (14.7%; *n* = 16 128). Diabetes (9.6%; *n* = 4220) and hypertension (55.9%; *n* = 24 432) prevalence were lowest in the cohort of patients with unmeasured uACR (Table 1).

### Cross-sectional summary of patient distribution by KFRE score categories

The total number and proportion of patients with CKD stages 3–5 and those with a calculable KFRE score varied by year: 17 880 (32.6%) in 2018; 22 229 (34.8%) in 2019; 14 854 (27.1%) in 2020; 19 225 (29.8%) in 2021; 21 586 (33.6%) in 2022; and 15 821 (34.7%) in 2023 (see Supplementary Table S2). The average proportion with KFRE score <5% was 89.2%; KFRE score 5%–20% was 7.7%; and KFRE score >20% was 3.1%. Annual proportions of patients across each KFRE category were generally stable across the study period.

The number and proportion of patients with a KFRE score >5% were 1657 in 2018 (9.3%), 2168 in 2019 (9.8%), 1691 in 2020 (11.4%), 2210 in 2021 (11.5%), 2425 in 2022 (11.2%), and 1828 in 2023 (12.6%), with an average of 11% over the study period (see Supplementary Table S2).

### Summary of crude outcomes by KFRE score categories

In total, there were 71 383 patients with CKD stages 3–5 with follow-up of 5–6 years; 30 197 (42.3%) had uACR measured in the index year (Table 2). Crude (unadjusted) mortality/RRT rates were 19.7%/0.1% for KFRE <5%; 35.9%/2.0% for KFRE 5%–20%, and 40.4%/20.1% for KFRE >20%. There were 2878 (9.5%) patients with a KFRE score >5% who could be eligible for referral to nephrology. A summary of results for disaggregated years 2018 and 2019 can be found in Supplementary Table S2.

**Table 2.** Proportions of events (death or RRT) for patients with CKD stages 3–5 across KFRE score categories in years 2018 and 2019 (aggregated) with a minimum follow-up period of 5 years

Category	2018 and 2019 aggregated
**Total complete cases CKD stages 3–5 (with and without uACR),** ***N***	71 383
**Total complete cases CKD stages 3–5 without uACR, *n***	41 186 (57.7)
Death, *n* (%)	8264 (20.1)
RRT, *n* (%)	446 (1.1)
**Total complete cases CKD stages 3–5 with uACR, *n* (%)**	30 197 (42.3)
**KFRE <5%**	27 319 (90.5)
Death	5390 (19.7)
RRT	32 (0.1)
**KFRE 5%–20%**	2076 (6.9)
Death	746 (35.9)
RRT	41 (2.0)
**KFRE >20%**	802 (2.7)
Death	324 (40.4)
RRT	161 (20.1)
**Total with a KFRE score >5%**	2878 (9.5)
Follow-up period, years	5–6

Summary of patients with CKD stages 3–5 and a calculable KFRE score (measured uACR) aggregated for years 2018 and 2019, which had ≥5 years study follow-up. Year 2018 had incomplete data (data began March 2020). The proportion of patients in KFRE score categories is based on total complete cases with uACR/total number of patients in that KFRE score category. Proportion of patients who died is based on total number of patients within each KFRE score category/total number of deaths in that KFRE score category. Proportion of patients who experienced RRT is based on total number of patients within each KFRE score category/total number of patients who experienced RRT within that KFRE score category. CKD = chronic kidney disease. KFRE = kidney failure risk equation. RRT = renal replacement treatment. uACR = urine albumin-to-creatinine ratio.

### Predictors of kidney failure and death

#### Kidney failure

Patients with a KFRE score of 5%–20% had ~21 times greater relative risk of RRT than those with a KFRE score <5% (95% confidence interval [CI] = 12.8 to 35.0; *P*<0.0001). Patients with a KFRE score >20% had ~200 times greater relative risk of RRT than those with KFRE scores <5% (95% CI = 130 to 318; *P*<0.0001) (Table 3).

**Table 3.** Multinomial regression of relative risk of RRT versus no event and death versus no event for patients with CKD stages 3–5

**Predictor**	Final multinomial regression model	
	**RRR (95% CI)**	*P*-value
**Outcome: RRT versus alive — no event**		
Intercept	0.001 (0.001 to 0.002)	**<0.0001**
KFRE category 5%–20%	21.1 (12.8 to 35.0)	**<0.0001**
KFRE category >20%	203.0 (130.0 to 318.0)	**<0.0001**
Asian/Asian British ethnicity	1.93 (1.27 to 2.94)	**0.002**
Black/Black British ethnicity	2.13 (1.17 to 3.85)	**0.01**
Mixed ethnicity	3.81 (1.42 to 10.4)	**0.009**
Other ethnic groups	1.61 (0.84 to 3.09)	0.15
IMD decile	0.98 (0.93 to 1.04)	0.53
Diabetes	1.11 (0.79 to 1.56)	0.53
Hypertension	0.99 (0.66 to 1.48)	0.97
Osteoporosis	0.83 (0.31 to 2.18)	070
Coronary heart disease	0.59 (0.37 to 0.93)	**0.02**
Heart failure	0.74 (0.35 to 1.56)	0.42
Stroke	0.51 (0.22 to 1.19)	0.12
Transient ischaemic attack	0.46 (0.14 to 1.49)	0.19
Autosomal dominant polycystic kidney disease	2.69 (1.52 to 4.75)	**0.0007**
Glomerulonephritis	1.74 (0.82 to 3.70)	0.15
Kidney stones	0.52 (0.18 to 1.48)	0.22
Acute kidney injury	1.35 (0.91 to 2.01)	0.13
Schizophrenia	0.89 (0.25 to 3.15)	0.86
Eating disorder	0.98 (0.27 to 3.58)	0.97
**Outcome: death versus alive — no event**		
Intercept	0.139 (0.128 to 0.152)	**<0.0001**
KFRE category 5–20%	2.27 (2.05 to 2.52)	**<0.0001**
KFRE category >20%	3.85 (3.25 to 4.56)	**<0.0001**
Asian/Asian British ethnicity	0.66 (0.58 to 0.75)	**<0.0001**
Black/Black British ethnicity	0.30 (0.23 to 0.40)	**<0.0001**
Mixed ethnicity	0.65 (0.41 to 1.02)	0.06
Other ethnic groups	5.07 (4.65 to 5.51)	**<0.0001**
IMD decile	0.96 (0.95 to 0.97)	**<0.0001**
Diabetes	1.15 (1.08 to 1.23)	**<0.0001**
Hypertension	1.20 (1.12 to 1.29)	**<0.0001**
Osteoporosis	1.91 (1.71 to 2.12)	**<0.0001**
Coronary heart disease	1.54 (1.44 to 1.65)	**<0.0001**
Heart failure	2.12 (1.94 to 2.33)	**<0.0001**
Stroke	1.75 (1.59 to 1.93)	**<0.0001**
Transient ischaemic attack	1.37 (1.22 to 1.54)	**<0.0001**
Autosomal dominant polycystic kidney disease	1.10 (0.86 to 1.42)	0.43
Glomerulonephritis	0.38 (0.22 to 0.66)	**0.0005**
Kidney stones	0.72 (0.60 to 0.87)	**0.0007**
Acute kidney injury	1.13 (1.04 to 1.22)	**0.003**
Schizophrenia	1.46 (1.17 to 1.82)	**0.0007**
Eating disorder	1.55 (1.24 to 1.94)	**0.0001**

Reference categories: KFRE category = KFRE <5%; ethnicity = White ethnicity; IMD decile = a one unit increase in IMD decile; and diagnoses = absence of diagnosis. CKD = chronic kidney disease. IMD = Index of Multiple Deprivation. KFRE = kidney failure risk equation. RRR = relative risk ratio. RRT = renal replacement treatment.

Patients of Asian and Black ethnicities had approximately double the relative risk of RRT (RRR 1.93, 95% CI = 1.27 to 2.94, *P* = 0.002; RRR 2.13, 95% CI = 1.17 to 3.85, *P* = 0.01, respectively) than White ethnicity. Patients of mixed ethnicity had approximately four times the relative risk of RRT (RRR 3.81, 95% CI = 1.42 to 10.4, *P* = 0.009). CHD was associated with a 41% lower relative risk of RRT (RRR 0.59, 95% CI = 0.37 to 0.93, *P* = 0.02) whereas autosomal dominant polycystic kidney disease was associated with ~2.5 times increased relative risk of RRT (RRR 2.69, 95% CI = 1.52 to 4.75, *P* = 0.0007) (Table 3).

#### Death

Patients with KFRE score of 5%–20% had ~2 times greater relative risk of death than those with a KFRE score <5% (95% CI = 2.05 to 2.52, *P*<0.0001); and patients with a KFRE score >20% had ~4 times greater relative risk of death than those with a KFRE score <5% (95% CI = 3.25 to 4.56, *P*<0.0001) (Table 3).

Patients of Asian and Black ethnicity had significantly lower relative risk of death (RRR 0.66, 95% CI = 0.58 to 0.75, *P*<0.0001; RRR 0.30, 95% CI = 0.23 to 0.40, *P*<0.0001, respectively) than White ethnicity. However, patients of other ethnic groups had ~5 times greater relative risk of death (RRR 5.07, 95% CI = 4.65 to 5.51, *P*<0.0001). A one decile increase in IMD was associated with a 4% lower relative risk of death (RRR 0.96, 95% CI = 0.95 to 0.97, *P*<0.0001). Diabetes, hypertension, osteoporosis, CHD, heart failure, stroke, transient ischaemic attack, AKI, schizophrenia, and eating disorder were all associated with statistically significantly higher relative risk of death. Glomerulonephritis and kidney stones were associated with significantly lower relative risk of death (Table 3).

### Summary of adjusted outcomes by KFRE score category

Using adjusted probabilities from the multinomial regression model a KFRE score of <5% was associated with a 0.1% probability of RRT (95% CI = 0.1 to 0.2), 11.9% probability of death (95% CI = 11 to 13), and 87% probability of no event (95% CI = 86 to 88). A KFRE score of 5%–20% was associated with 2% probability of RRT (95% CI = 1 to 3), 23% probability of death (95% CI = 20 to 27), and 75% probability of no event (95% CI = 70 to 78). KFRE score >20% was associated with 14% probability of RRT (95% CI = 8 to 23), 29% probability of death (95% CI = 25 to 36), and 57% probability of no event (95% CI = 48 to 63) (Table 4). As KFRE score increased, so did the probability of RRT. The probability of death not only increased with KFRE category but was statistically significantly greater than the probability of RRT within each category.

**Table 4.** Predicted probabilities of outcomes by KFRE category — derived from multinomial regression model (Table 3)

KFRE category	2018 and 2019 aggregated		
	Predicted probabilities (95% CI)		
	Alive — no event	RRT	Death
<5%	0.87 (0.86 to 0.88*)*	0.001 (0.001 to 0.002)	0.119 (0.110 to 0.130)
5–20%	0.75 (0.70 to 0.78)	0.02 (0.01 to 0.03)	0.23 (0.20 to 0.27 )
>20%	0.57 (0.48 to 0.63)	0.14 (0.08 to 0.23)	0.29 (0.25 to 0.36)

Predicted probabilities and 95% CIs of experiencing no event, RRT, or death across KFRE categories from a final multinomial regression model where all *P*-values corresponding to KFRE categories were statistically significant (*P*<0.0001). Predicted probabilities based on following reference categories: ethnicity = White; IMD = 1; and diagnoses = no diagnoses at study start. KFRE = kidney failure risk equation. RRT = renal replacement treatment.

Given the statistically significant associations of ethnicity, IMD, and clinical comorbidities on relative risk of death and RRT (Table 3), plotting predicted probabilities according to interactions between these covariates revealed important patterns in the distribution of outcomes among KFRE categories. The probability of death exceeded RRT across each KFRE category for patients of White ethnicity, regardless of IMD decile (1, 5, or 10) (Table 5 and Figure 1). Additionally, patients of White ethnicity had a greater probability of death than patients of Asian or Black ethnicities, across each KFRE category. For patients of Asian or Black ethnicities the probability of death was greater than RRT in KFRE categories <5% and 5%–20% (across all IMD deciles); however, in KFRE category >20%, the probability of RRT was greater than death. Moreover, for patients of Black ethnicities, the difference in probability between RRT and death was greater for KFRE category >20%, than for Asian patients.

**Table 5.** Summary of predicted probabilities of outcomes by KFRE categories, ethnicity, and IMD deciles (1, 5, and 10)

Predicted probabilities of outcomes				
IMD decile	KFRE category	Alive — no event	Death	RRT
**White ethnicity**				
1	<5	0.842	0.157	0.001
1	5–20	0.689	0.291	0.019
1	>20	0.502	0.359	0.138
5	<5	0.860	0.138	0.001
5	5–20	0.719	0.262	0.019
5	>20	0.533	0.330	0.136
10	<5	0.881	0.117	0.001
10	5-20	0.753	0.228	0.018
10	>20	0.572	0.294	0.133
**Asian or Asian British ethnicity**				
1	<5	0.888	0.109	0.002
1	5–20	0.750	0.208	0.041
1	>20	0.499	0.235	0.266
5	<5	0.902	0.095	0.002
5	5–20	0.774	0.185	0.039
5	>20	0.526	0.214	0.259
10	<5	0.917	0.080	0.002
10	5–20	0.802	0.160	0.037
10	>20	0.559	0.189	0.251
**Black or Black British ethnicity**				
1	<5	0.944	0.053	0.002
1	5–20	0.841	0.107	0.051
1	>20	0.555	0.120	0.325
5	<5	0.951	0.046	0.002
5	5–20	0.857	0.094	0.048
5	>20	0.578	0.107	0.314
10	<5	0.959	0.038	0.002
10	5–20	0.875	0.080	0.044
10	>20	0.606	0.093	0.299

Presence of diabetes and hypertension were held constant. IMD = Index of Multiple Deprivation. KFRE = kidney failure risk equation. RRT = renal replacement treatment.

**Figure 1.**
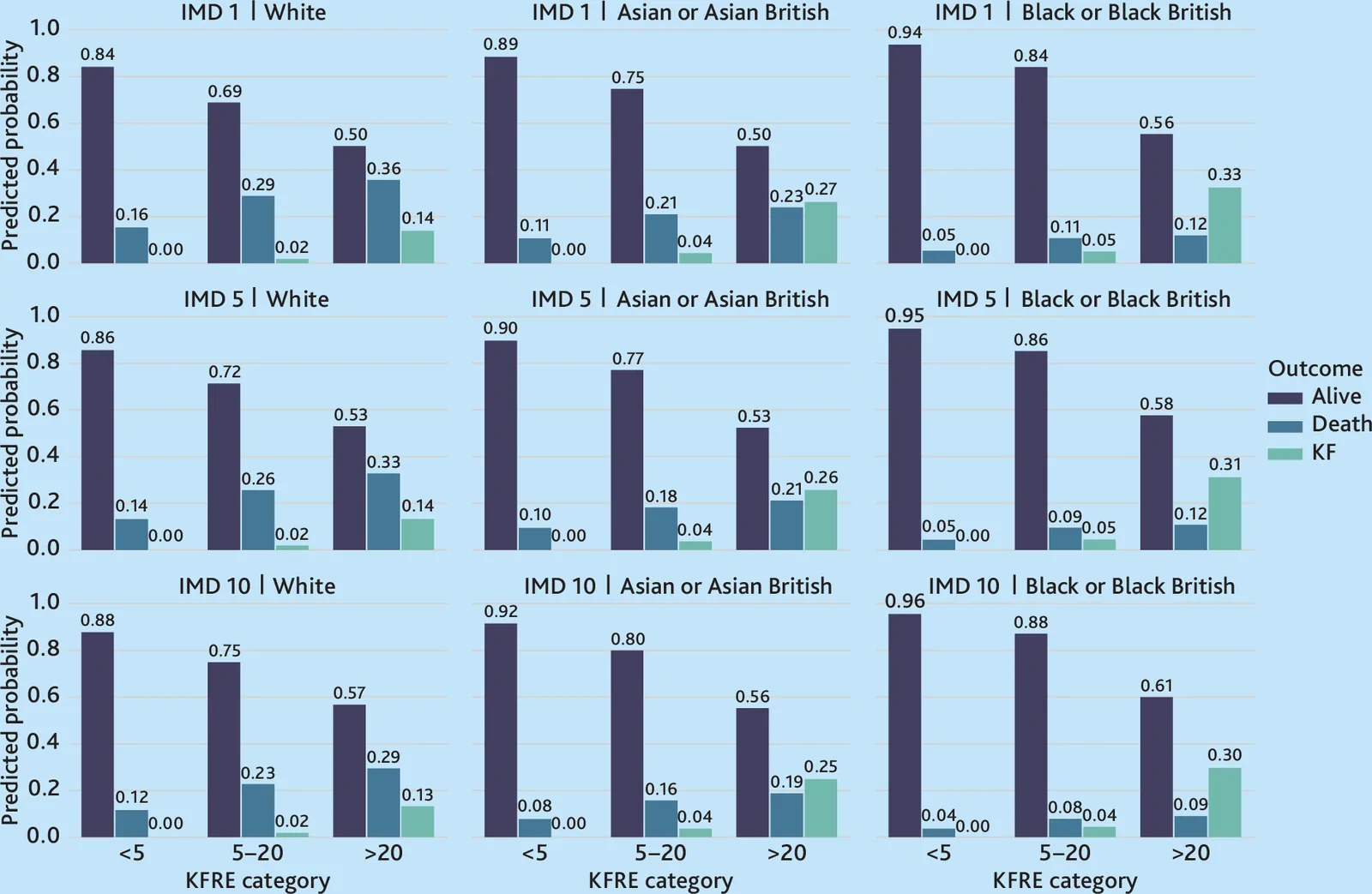
Predicted probabilities of outcomes by KFRE category, ethnicity, and IMD, where presence of diabetes and hypertension is held constant. Predicted probabilities ascertained from final model (Table 5) of outcomes (alive — with no event; death; and kidney failure [KF]) by KFRE category, ethnicity, and IMD deciles (1, 5, and 10), with diabetes and hypertension held constant. Probabilities rounded to 2 decimal places. IMD = Index of Multiple Deprivation. KFRE = kidney failure risk equation.

## Discussion

### Summary

KFRE score was a statistically significant predictor of RRT and death. The probability of death was greater than the probability of RRT for patients of White ethnicity across each KFRE category. However, for patients of Black and Asian ethnicities, the probability of death was greater than RRT for KFRE categories <5% and 5%–20%, but lower for KFRE category >20%. The number and proportion of patients with CKD stages 3–5 and a KFRE score >5% who could be referred to nephrology services were 1657 in 2018 (9.3%), 2168 in 2019 (9.8%), 1691 in 2020 (11.4%), 2210 in 2021 (11.5%), 2425 in 2022 (11.2%), and 1828 in 2023 (12.6%); this was an average of 11% (see Supplementary Table S2).

At the patient level, the KFRE is a useful tool for predicting an individual’s risk of RRT. At the population level, the authors show that it is a useful tool for quantifying the number of patients who could be eligible for referral to nephrology services.^6^ However, its blindness to competing risk of death^5^ is a limitation for primary care clinicians to be aware of and a prompt to consider additional risk prediction tools to facilitate earlier cardiovascular risk management.

### Strengths and limitations

Key strengths of this study include that it was a large contemporary primary care sample of 109 543 patients with coded and uncoded CKD; all eGFRs were recalculated using the CKD-EPI 2021 equation;^9^ regression analyses was adjusted for an extensive list of comorbidities and renal specific diagnoses. A further strength was its contribution to the limited evidence base of mental health disease prevalence in CKD. The following limitations exist: complete cases were analysed due to computational limitations within the GMCR virtual environment to run multiple imputation for missing data; missing data regarding uACR was high (from 65.2% in 2019 to 72.9% in 2020), which may limit generalisability to patients with unmeasured uACR. Further limitations were that multinomial regression does not take into account the time-to-event nature of the data, which may limit the robustness of reported probabilities; kidney failure was defined using diagnostic codes, which excludes patients receiving conservative care who may have higher risk of death; analysed data included data during and after the COVID-19 pandemic that impacted investigation and management of CKD in primary care.^5^

### Comparison with existing literature

The present study showed that the KFRE overestimated the risk of RRT (0.1% [95% CI = 0.1 to 0.2] for KFRE <5%; 2% [95% CI = 1 to 3] for KFRE 5%–20%; and 14% [95% CI = 8 to 23] for KFRE >20%), which has been observed elsewhere.^3,7,11^ Importantly, the risk of death generally exceeded RRT across KFRE categories, which is observed by Liu *et al*.^7^ The high competing risk of death likely reflects the high prevalence of cardiovascular disease, multimorbidity, and older age of the primary care population in the present study. Accordingly, the lower relative risk of RRT observed among adults with coronary heart disease (RRR for RRT versus alive = 0.59, 95% CI = 0.37 to 0.93, *P* = 0.02) reflects the higher burden of cardiovascular disease in this group, which increases the likelihood of death (RRR in patients with coronary heart disease for death versus alive = 1.54, 95% CI = 1.44 to 1.65, *P*<0.0001) before RRT becomes necessary. Moreover, older adults are more likely to be treated with conservative kidney management rather than RRT.^12^ Interestingly, patients of Black and Asian ethnicities with a KFRE score >20% had a higher risk of RRT than death across IMD deciles, which may reflect faster progression of CKD due to hypertensive and diabetic nephropathy, and APOL1-related kidney disease in patients of Black ethnicity.^12^ Addressing overestimation of RRT, KDpredict^7^ — an alternative risk prediction tool — simultaneously predicts the risk of RRT and death up to 5 years, which can help GPs tailor care towards the outcome with the greater risk.

To the authors’ knowledge, this article presents the first estimate (11% average) of patients with CKD stages 3–5 with a KFRE score >5% who could be eligible for referral to nephrology services from a large contemporary English primary care cohort. The estimate from the present study is between existing estimates: 1% of patients with CKD stage 3 had a KFRE score >5% in Welsh primary care^13^; and 21.9% of patients with CKD stages 3–5 had a KFRE score >3% in UK primary care.^14^ Despite NICE guidance^6^ recommending the use of the KFRE since 2021, there is limited evidence on the implementation of the KFRE in primary care and how it is impacting referrals to nephrology, though early evaluations, such as in the LUCID project,^15^ suggest it may optimise referral appropriateness by expediting referrals of high-risk cases while reducing unnecessary referrals. Lower deprivation was associated with lower risk of death (RRR 0.96, 95% CI = 0.95 to 0.97) — shown before in a CKD stages 1–5 secondary care population^16^ (odds ratio 0.62; 95% CI = 0.32 to 0.94).

### Implications for research and practice

The higher probability of RRT than death for patients of Black and Asian ethnicities with a KFRE score >20%, alongside a general overestimation of risk of RRT using KFRE, is important because NICE guidance does not consider ethnicity or the competing risk of death for referral recommendations.^6^ This may inadvertently result in over-referral of older patients of White ethnicity, while other patients (for example, patients of Black and Asian ethnicity) at higher risk of RRT are not prioritised for early intervention. In practice, GPs can use both tools: the KFRE — despite its tendency to overestimate risk — has been validated in a large diverse primary care population in England^3^ and is recognised by NICE, therefore it can be used to estimate risk to guide referrals; KDpredict^7^ can be used to contextualise the estimate by providing the competing risk of death. Future iterations of the KFRE could be improved by modelling the competing risk of death to better align predicted risks with real-world outcomes. Together, both prediction models can improve clinical management, shared decision making, and equity of care. Moreover, the availability of medications capable of reducing the risk of kidney disease progression and cardiovascular mortality (sodium glucose cotransporter-2 inhibitors^17–19^ and glucagon-like peptide-1 receptor agonists)^20,21^ emphasise the need to identify patients at greatest risk.^5,22^

Albuminuria testing is central to accurate kidney disease risk prediction, which was unmeasured in 43 742 (39.9%) patients with CKD stages 3–5 during the presented study’s 6-year period. This finding is broadly consistent with other UK^5,15^ and international primary care studies.^23,24^ Incomplete testing reflects factors including a lower prioritisation of uACR testing in UK primary care since the removal of financially incentivised CKD care through the Quality and Outcomes Framework in 2015, a lack of clinician awareness regarding the prognostic value of albuminuria, and competing clinical priorities.^5^ Clinicians and policymakers must review and strengthen incentives, such as embedding KFRE-linked alerts or quality indicators in electronic health records (EHRs), to increase albuminuria testing in patients at risk of and with CKD.^5,25^

Knowing the proportion of all patients with CKD stages 3–5 potentially eligible for referral (~11%) is useful for developing referral pathways and planning nephrology services including access to RRT. Such a figure would be useful given ~40% of patients with CKD stages 3–5 are uncoded in primary care and possibly unknown to both primary and secondary care.^5,16^

## Data

Researchers can apply to use the same Greater Manchester Care Record data through an application to the GMCR board at the following website: https://healthinnovationmanchester.com/contact.
